# Rehabilitation in People Living with Glioblastoma: A Narrative Review of the Literature

**DOI:** 10.3390/cancers16091699

**Published:** 2024-04-27

**Authors:** Anna Zanotto, Rebecca N. Glover, Tobia Zanotto, Florien W. Boele

**Affiliations:** 1 Department of Occupational Therapy Education, School of Health Professions, University of Kansas Medical Center, 3901 Rainbow Blvd, Kansas City, KS 66160, USA; rglover2@kumc.edu (R.N.G.); tzanotto@kumc.edu (T.Z.); 2 Patient Centred Outcomes Research Group, Leeds Institute of Medical Research at St James’s, University of Leeds, Leeds LS2 9JT, UK; 3 Academic Unit of Health Economics, Leeds Institute of Health Sciences, University of Leeds, Leeds LS2 9JT, UK

**Keywords:** glioblastoma, neuro-oncology, quality of life, rehabilitation, occupational therapy, physical therapy, cognitive rehabilitation

## Abstract

**Simple Summary:**

Glioblastoma is the most common primary malignant brain tumor. Preliminary data suggest that rehabilitation can have positive effects on patients with glioblastoma. However, there are unique challenges for clinicians working with this population, including limited life expectancy and/or rapid neurological deterioration. The aim of this article is to review the literature on the rehabilitation of adults with glioblastoma. The reviewed literature suggests that rehabilitation can be beneficial for improving the quality of life of adult patients with glioblastoma. We summarize the evidence regarding healthcare professionals’ and patients’ perspectives on the use of supportive care services. We conclude that there is a need for the design of effective rehabilitation programs for patients with glioblastoma, as well as for the development of glioblastoma-specific clinical guidelines for rehabilitation practitioners.

**Abstract:**

Glioblastoma is the most common primary malignant brain tumor. While preliminary data point to the positive effects of rehabilitation for patients with glioblastoma, there are unique challenges for clinicians working with this population, including limited life expectancy and/or rapid neurological deterioration. The aim of this article is to review the literature on rehabilitation of adults with glioblastoma, including the feasibility of interventions, their effectiveness, as well as the current clinical practice. The reviewed literature suggests that rehabilitation has been found beneficial for improving the functional prognosis and quality of life of adults with glioblastoma and is desired by patients. We summarize the qualitative evidence regarding healthcare professionals’ and patients’ perspectives on the use of supportive care services. We conclude there is a need for the design of effective rehabilitation programs for patients with glioblastoma, as well as for the development of glioblastoma-specific clinical guidelines for rehabilitation practitioners.

## 1. Introduction

Glioblastoma is a malignant brain tumor with a poor prognosis and a median survival of less than 2 years [1]. It is the most common primary malignant brain tumor and represents 57% of all gliomas and 48% of all primary malignant central nervous system (CNS) tumors [2]. Individuals diagnosed with glioblastoma are faced with a progressive decline of neurologic function and significant symptoms both from the tumor itself but also due to the toxicities from therapy [1]. Negative implications on daily functioning, usual life activities, and quality of life are common, and all combined can have a devastating impact on patients, caregivers, and families [3,4]. Limited effective treatments exist. The optimal treatment has remained the same since 2005 and consists of surgery to remove as much of the tumor as feasible, followed by a 6-week course of chemo- and radiotherapy (delivered together), and followed by six cycles of monthly chemotherapy [5]. We know that chemotherapy is most effective in those who have a specific molecular marker (MGMT promoter methylation, present in ~40% of glioblastomas) [6,7]. This optimal treatment extends survival by a number of months: median survival is 8 months regardless of what kind of treatment is given, and 14 months for those receiving optimal treatment. However, only about 60% of patients are eligible for this optimal treatment (due to, e.g., severe cognitive issues or frailty), and only about a third of those who start the regimen finish it [8]. Nearly half (46.3%) of people diagnosed with glioblastoma die in the year after diagnosis [9]. It is worth noting that the new 5th edition of the World Health Organization (WHO) classification of CNS tumors introduced changes to the diagnostic criteria of glioblastoma [10]. The role of molecular diagnostics was highlighted with integrated diagnoses now being based on histopathology and molecular information and tumors being grouped based on genetic alterations [11]. According to the new classification, the same disease can have a different prognosis depending on the mutational pattern [12]. Recent years have also seen developments of the chimeric antigen receptor (CAR) T cell therapy, a type of immunotherapy, which to date have had limited effectiveness in glioblastoma [13]. While early results did not show clinical benefit, it is a rapidly expanding area of investigation and several CAR T cell therapies are currently being tested in clinical trials for glioblastoma [13].

## 2. Overview of Rehabilitation for Glioblastoma

Due to a high rate of disabling sequelae, including cognitive deficits, weakness, and visual perceptual changes, rehabilitation constitutes an important part of healthcare for patients with high-grade glioma [14]. While the primary purpose of rehabilitation is optimizing function, other concerns are also addressed including symptom management, quality of life, social and environmental factors (family, work, etc.), as well as knowledge of the underlying medical situation and illness trajectory [14]. Thus, there is an overlap with supportive and palliative care services. In a review of the management of glioblastoma, Tan et al. [1] highlighted that supportive care is of paramount importance in the multimodal treatment approach and important from a holistic patient care perspective [1]. Supportive care includes the management of seizures, fatigue, pain, venous thromboembolism (VTE), mood and behavioral disorders, and cognitive rehabilitation [1,3]. The importance of early and active involvement in palliative care is increasingly recognized as crucial for improving quality of life and reducing symptom burden [3]. Rehabilitation has been defined as “a problem-solving educational process aimed at reducing disability and handicap (participation) experienced by someone as a result of disease or injury” [15]. In the current review, we conceptualize rehabilitation as encompassing occupational therapy (OT), physical therapy (PT), and cognitive rehabilitation. However, we recognize that there is a broader range of healthcare professionals who can be involved in rehabilitation, including nursing, social work, psychology, and other allied health professionals.

Rehabilitation has been found beneficial for improving the functional prognosis and quality of life of adults with glioblastoma [16,17,18] and is desired by patients [19]. An early comparative study of inpatient rehabilitation among patients with brain tumors and patients with traumatic brain injury showed functional improvements for both groups [20]. Inpatient rehabilitation has been shown to have favorable effectiveness in people diagnosed with brain tumors, with functional improvements and homegoing rates similar to those of individuals with other neurologic conditions, such as stroke or traumatic brain injury [14,20,21,22,23,24]. People living with diseases characterized by poor prognosis are commonly eager to connect with everyday life [14]. In such cases, hope might be related to having fulfilling life experiences rather than to the prospect of a cure [25]. This is particularly relevant in the context of glioblastoma. Yet, there is minimal literature available to guide recommendations when working with individuals with glioblastoma in a rehabilitation setting [26]. It is recognized that these individuals can have limited awareness or acceptance of their diagnosis and/or prognosis [27], presenting challenges for occupational therapists, physical therapists and speech pathologists working with these individuals. Alongside these issues, referral to and uptake of rehabilitation services lag behind [28].

The majority of the available literature examining rehabilitation in neuro-oncology relates to high-grade gliomas. Glioblastoma is the most common high-grade glioma [29], and thus conclusions from this literature may be considered to inform recommendations when providing rehabilitation services for individuals living with glioblastoma. A Cochrane literature review published in 2013 [26] examined the effectiveness of multidisciplinary rehabilitation in adults with primary brain tumors. The authors were unable to identify any published randomized controlled trials or controlled clinical trials of multidisciplinary rehabilitation interventions in adults after treatment for primary brain tumors. However, based on 12 observational studies, they tentatively concluded that there is very low-level certainty of evidence to suggest that rehabilitation may improve functional outcomes, vocation, and quality of life [26]. In an update published in 2015 [30], one study was identified and added [31], reinforcing the preliminary conclusions that higher-intensity outpatient rehabilitation can reduce short- and long-term disability in people with brain tumors as compared to standard care. A restricted literature search performed in 2019 by the Cochrane Review group did not identify any new potentially relevant studies to change these conclusions. There is a need to review literature specific to glioblastoma diagnosis. Overall, the findings on the success of rehabilitation interventions for individuals with gliomas, including best practices, settings, type, intensity, and duration of rehabilitation are inconclusive [17]. However, it has been suggested that therapy is feasible, well-tolerated, non-detrimental and can improve disability and quality of life [17]. Published literature to date, from multiple groups across the United States and Europe, seems to suggest that when working with individuals with high-grade gliomas rehabilitation should occur early, using a multidisciplinary team approach to provide individually tailored services, and addressing the functional impairments affecting these individuals [16,17,32,33]. Typically, rehabilitation includes physical therapy, occupational therapy, cognitive rehabilitation, and psychological support for patients with a glioma and their caregivers [17]. The timing of rehabilitation can vary among individuals, from early after surgery to weeks after active medical management, and can be completed in inpatient, outpatient, and home-based settings [17]. When working with individuals with a high-grade brain tumor, especially glioblastoma, it is imperative to address the psychosocial impact on these individuals and their loved ones by providing supportive care and education [16,34]. Aiming to improve quality of life should be considered a top priority in therapy when working with this population. Providing psychological support for patients and their caregivers throughout the disease and treatment trajectory, when taking special consideration for their needs and preferences, has been found to be valuable in improving perceived quality of life [16].

The subsequent sections of this narrative review of literature aim to provide an overview of selected research and practice recommendations for rehabilitation in adults living with glioblastoma more specifically. The different elements of rehabilitation that will be covered include occupational therapy, physical therapy, and cognitive rehabilitation.

## 3. Occupational Therapy Rehabilitation

Occupational therapy is an essential part of the multidisciplinary team for addressing the unique motor, cognitive and psychosocial needs of individuals living with glioblastoma. A central premise of occupational therapy is that health and wellbeing, and perceived quality of life are influenced by participation in everyday life occupations [33]. When working with patients with glioblastoma, occupational therapists collaborate with the patient and their loved ones to determine goals, identify limiting factors impeding occupational performance, and use a client-centered approach to improve function or adapt to loss of function [33]. Occupational therapists use restorative and compensatory intervention approaches, and provide education tailored to the needs and preferences of the patient and their loved ones [33]. When providing education and psychological support, it is recommended that it includes the family and/or caregivers [34]. Occupational therapists are commonly faced with challenges and ethical dilemmas when setting client-centered goals for individuals with glioblastomas, due to a lack of insight or understanding of the prognosis commonly seen within this population [27].

Although there are emerging efforts examining best rehabilitation practices for individuals with brain tumors, there is currently limited and inconclusive evidence to provide recommendations for occupational therapy rehabilitation protocols for this population. The strength of evidence is low, due to small sample sizes, poor longitudinal follow-up, lack of control groups and the use of heterogenous groups, making it difficult to compare outcomes [16]. Research aimed at identifying best practices for providing education and supportive care, as well as developing recommendations for goal setting with this patient population, could have a positive impact on occupational therapy practice when providing rehabilitative services for individuals living with glioblastoma and their loved ones.

We were only able to identify two published rehabilitation trials that included occupational therapy as one of the components: one feasibility study [35] and one randomized controlled trial [36]. Neither of the studies specifically indicated whether participants had glioblastoma; however, a large proportion had a WHO grade IV glioma (75% out of 24 participants [35] and 63% out of 32 participants [36], respectively). Both studies included physical therapy as the other component of the intervention, therefore it is difficult to separate the effects of occupational therapy alone. The first study concluded that an intensive rehabilitation intervention combining physical therapy and occupational therapy is feasible and safe [35]. While the results of the RCT showed no effectiveness of the intervention regarding improvements in patients’ overall quality of life, there were positive findings for secondary outcomes of health-related quality of life domains (cognitive functioning and fatigue), symptomatology, and objectively measured functional performance as compared to the control group [36]. This suggests that including occupational therapy in the rehabilitation of patients with glioma can yield promising results. Further research is needed to examine the effectiveness of occupational therapy interventions and to indicate how they could be improved in order to provide greater benefit for patients with glioblastoma, specifically.

Hammill et al. [37] conducted a qualitative study with people with brain cancer looking at the impact of disease on occupational participation and the role occupational therapy can play in enhancing patient participation. The authors reported that the role of occupational therapy was often misunderstood by the participants, and patients were not referred. They also found that occasionally the focus of care did not match patients’ expectations, which led to their dissatisfaction with the services provided [37]. McCartney et al. [38] also reported that among clinicians interviewed (General Practitioners, clinical nurse specialists, and radiographers) there was a lack of understanding of the role of occupational therapists in the rehabilitation of patients with high-grade brain tumors. They reported a lack of understanding of the relevance of rehabilitation for this group of patients and, in particular, a lack of knowledge about brain tumors and their management [38].

To provide the most comprehensive rehabilitative care for individuals with glioblastoma, it is necessary to utilize a multidisciplinary team approach [39]. There may be a degree of overlap in how various disciplines address the impairments of body structures and functions, and activity limitations of the individual living with glioblastoma. This would require the therapy team to discuss how each discipline will address the client and family goals through their unique lens, with the ultimate goal of improving function, participation in meaningful activities and improving perceived quality of life. Occupational therapists use a variety of approaches to address the broad spectrum of symptoms experienced by individuals living with glioblastoma, including upper extremity impairments, impaired ability to perform activities of daily living (ADLs) and instrumental activities of daily living (IADLs), visual impairments, cognitive impairments, impaired functional mobility, and decreased activity tolerance [39]. Occupational therapists take into consideration the client’s understanding of their diagnosis and prognosis when developing a plan of care and may adapt it accordingly. As a result of a glioblastoma diagnosis, many individuals receiving treatment experience side effects, often including significant fatigue and changes to their cognitive function [14]. Occupational therapists use a client-centered approach to continually assess the individual and adapt the plan of care based on how the individual is presenting. Education is important for all stages of the disease process and should be tailored to the client as well as including their loved ones. Occupational therapists incorporate education on sleep hygiene, and the benefits of having daily structure and routine to help manage some of the symptoms, and to improve participation in meaningful activities.

## 4. Physical Therapy Rehabilitation

Despite the general consensus that physical therapy interventions are beneficial as part of individualized palliative care plans for people with glioblastoma [40], there are currently only a few published reports and specific recommendations are lacking. Yet, there are several theoretical reasons why physical therapy may enhance the health span of people living with glioblastoma. For instance, physical rehabilitation may support better mobility which is associated with better survival outcomes. In a recent study of glioblastoma acute rehabilitation inpatients, lower levels of inpatient mobility, as assessed through the Activity Measure for Post-Acute Care (AM-PAC) “6-Clicks”, were associated with a greater risk of transfer to the acute care service [41]. In turn, patients who underwent this unplanned transfer had a shorter survival compared to patients who were not transferred. Relatedly, higher levels of physical activity are associated with lower severity of brain cancer-specific concerns and higher quality of life in people living with a primary brain tumor [42].

In addition, people with glioblastoma have markedly lower levels of muscle strength compared to age- and sex-matched individuals living without a brain tumor [43]. While reduced muscle strength may represent a neurologic symptom, the chronic use of corticosteroids (prescribed to treat vasogenic edema and reduce intracranial pressure) combined with the neurotoxic side effects of chemotherapy is thought to exacerbate muscle wasting in this clinical population [44]. To counteract the side effects of medications, exercise-based interventions involving resistance training may represent a suitable strategy to restore muscle function and promote anabolic processes [45]. In this respect, multicomponent exercise programs involving resistance training and moderate-intensity aerobic exercise have been shown to be feasible, safe, and well-tolerated in people living with a glioblastoma [46,47,48,49]. In their study, Halkett et al. [47] also highlighted that people receiving chemoradiotherapy for glioblastoma perceived the exercise program to be beneficial for their physical and psychological health. Some potential barriers and concerns, such as managing symptoms and juggling exercise and treatment, were also reported [47]. Although common symptoms such as dizziness and fatigue may prevent or discontinue participation in exercise interventions, recent evidence from a case report study [50] as well as from a small-scale randomized controlled trial [51] suggest that even high-intensity exercise can be tolerated without adverse events by some people with glioblastoma. Additionally, the case study [50] highlighted that in some cases, despite the poor prognosis, patients can still participate in strenuous (and meaningful) sporting events such as running a marathon. These findings underscore the high heterogeneity of people living with this condition, which likely warrants an individualized approach to exercise-based rehabilitation, based on the individual’s goals but also in terms of preferences for exercise modalities that are perceived as meaningful. Table 1 summarizes selected research studies on physical therapy rehabilitation for patients with glioblastoma.

In summary, while the scientific evidence concerning the effectiveness of physical therapy interventions to manage symptoms and improve quality of life in people with glioblastoma remains scarce, preliminary findings strongly indicate that multicomponent exercise programs should be further investigated and considered when addressing the rehabilitation needs of people living with a glioblastoma. The results need to be interpreted with caution, as the data come from case studies or small-scale feasibility studies, and therefore limit the conclusions which can be drawn. However, these observations can be further bolstered by the numerous systematic reviews supporting the safety and effectiveness of exercise-based interventions in people living with cancer [52,53].

From a practical perspective, the role of physical therapy in people with a glioblastoma can be categorized into two main complementary approaches: (1) managing primary and secondary neurological symptoms, such as gait and balance impairments [54], through tailored programs of gait, balance, and lower extremity strengthening exercises [55]; (2) maximizing physiological function and resistance to stressors through moderate to vigorous regimens of multicomponent exercise training [48,50]. Importantly, maximizing physiological function would be fundamental at the pre-habilitation stages, namely before undergoing surgical interventions or chemoradiotherapy. Indeed, improving overall fitness is often associated with a better ability to withstand stressors, such as surgery or the side effects of chemoradiotherapy [56,57]. Considering the patient’s preferences in terms of exercise modality is also likely critical for ensuring optimal treatment adherence and higher enjoyment, which in turn could lead to a higher quality of life.

## 5. Cognitive Rehabilitation

People with glioblastoma are at high risk for developing cognitive deficits due to the presence and location of the tumor, but also due to side effects of surgery, radiotherapy, and chemotherapy [58,59]. In a longitudinal prospective study with 49 participants with glioblastoma undergoing surgery, Sinha et al. [60] found that before surgery, the participant risk of deficit was increased in five out of six cognitive domains as compared to normative groups (especially increased risks were to attention, memory, and perception). Two weeks after the surgery, when patients were discharged home, these risks significantly increased. Notably, for those participants who had a good enough performance status postoperatively to be able to undergo chemotherapy and radiotherapy at 4–6 weeks after surgery, the authors reported that the risks of cognitive deficits reduced and resembled those seen at the presurgical baseline [60]. These findings suggest that there are individual differences in cognitive performance postoperatively and there is a need for personalized cognitive rehabilitation in this population. 

Cognitive impairments can negatively affect health-related quality of life, and present barriers to care and reintegration into society [61]. Management of cognitive deficits in people with primary brain tumors may include cognitive rehabilitation and/or pharmacotherapy. For a comprehensive review, we direct the reader to a recently published systematic review of cognitive interventions for adults with brain tumors by Kirkman and colleagues [62]. Out of 35 studies included in their review, 9 included at least a proportion of participants with glioblastoma (ranging from 4.8% to 100% of the study sample). Kirkman et al. [62] included both randomized and nonrandomized studies; 14 studies reported on a pharmacological intervention and 21 on a nonpharmacological intervention. Examples of pharmacological agents that were associated with positive effects on cognition included memantine (NMDA receptor antagonist), donepezil (cholinesterase inhibitor), methylphenidate (CNS stimulant), modafinil (wakefulness-promoting agent), ginkgo biloba, and shenqi fuzheng. Among the nonpharmacological interventions, working memory training, goal management training, aerobic exercise, virtual reality training combined with computer-assisted cognitive rehabilitation, hyperbaric oxygen therapy, and semantic strategy training were found to be linked to better outcomes in cognitive domains [62]. Regarding the potential mechanisms of action, because of the high diversity of the nonpharmacological interventions, the pathogenic basis of action is also likely to be varied [62]. For example, attention or working memory retraining may involve brain plasticity. Physical exercise interventions may act to bring positive effects in the cognitive domain through increased cerebral blood flow and hippocampal neurogenesis, as well as changes in neurotransmitter release or arousal level [62]. Overall, caution must be exercised when interpreting these results, as most of the included studies were at moderate-to-high risk of bias. Most of the included studies had a number of methodological limitations and most did not report effect sizes, which then further limits the ability to interpret the strength of identified relationships. Moreover, it remains unclear to what extent the results can be observed beyond the end of the intervention. The authors called for greater collaboration between the research centers and the use of more uniform outcome measures [62]. Table 2 summarizes selected research studies on nonpharmacological interventions for cognitive function which included at least 30% of participants with glioblastoma in their sample.

Cognitive rehabilitation is usually delivered by speech and language pathologists, neuropsychologists, or occupational therapists. Assessment of cognitive function is important as knowing the capacity can facilitate adjustment and long-term planning [14]. The three clinical guidelines, the Australian Cancer Network (ACN) [63], the National Comprehensive Cancer Network (NCCN) [64], and the National Institute for Health and Clinical Excellence (NICE) [65] guidelines recommend a formal neuropsychological evaluation as the gold standard for assessment of neurocognitive function, as it can objectively and comprehensively characterize the cognitive, behavioral, and emotional issues related to the diagnosis, as well as identify cognitive strengths. A full assessment can contribute to the identification of the risk factors that are contributing to the patient’s neurocognitive difficulty and reduced function, but which may be treatable (e.g., depression or sleep disturbance). Evaluations also lead to providing patient-specific recommendations, including for example implementation of compensatory strategies in daily activities, or guidance around work accommodations [64]. Regular monitoring of cognitive and personality changes is also recognized as an important part of the patient’s continuing care [64,65]. ACN also recommends that as depression and anxiety can interfere with a patient’s capacity to make treatment decisions, it should be treated with a combination of psychotherapy and cognitive behavioral therapy, along with relaxation therapy or guided imagery to help deal with stressful situations [63].

**Table 2:** Summary of selected studies of nonpharmacological cognitive rehabilitation in people with glioblastoma.

Author	Study Design	Population	Intervention	Outcomes
Braun et al., 2021 [66]	Feasibility study	16 people with high- and low-grade glioma (30% people with glioblastoma).	5 weeks of 50 min of online CogMed Working Memory Training 5 days a week.	80% retention rate (16/20 enrolled patients completed the intervention). No adverse events. 69% adherence Moderate perceived degree of benefit Medium to large effects and significant increases in Wechsler Adult Intelligence Scale (WAIS) Digit Span and WMS Symbol Span were observed from baseline to post-training.
Hassler et al., 2010 [67]	Pilot study	11 people with high-grade glioma (63.6% people with glioblastoma).	10 weekly 90-min sessions of holistic mnemonic training.	Comparison of mean baseline and post-training group differences revealed nonsignificant improvements in all but one cognitive variable (Hopkins Verbal Learning Test (HVLT) Total Learning, mean difference = 4.0, *p* = 0.04).

Note. The studies were selected to represent nonpharmacological cognitive rehabilitation interventions with at least 30% of participants reported to have a glioblastoma diagnosis.

## 6. Multidisciplinary Approach

In addition to the evidence-based interventions used to rehabilitate sensory and motor deficits as a result of glioblastoma or medical treatment for glioblastoma, clinicians should utilize a multidisciplinary team approach that includes occupational therapy, physical therapy, speech therapy, and social work [14]. All disciplines should perform an initial evaluation to determine the individual’s understanding of their diagnosis; determine impairments of body structures and functions, and activity limitations; and identify client and family-centered goals. The team should then collaborate to determine the most appropriate plan of care to address the unique needs [39]. In some cases, despite the severity of the physical and sensory impairments that can be seen with this diagnosis, the therapy team may adapt their recommended plan of care to allow the individual to spend more time at home with their loved ones, participating in meaningful activities that will improve their perceived quality of life. The therapy team should also make referrals to other healthcare professionals such as nurses if there are medical needs related to the individual’s care; social workers to provide education about glioblastoma and to provide information about resources available; and psychology to help with coping and adjustment to their diagnosis. As there is limited evidence to guide best practices when working with this population, this leaves therapists to rely on their own clinical experience and to use literature supporting treatments for diagnoses that result in similar symptoms to guide their practice.

## 7. Palliative Care

Early referral to palliative care can improve the quality of life. According to the NICE, ACN, and NCCN guidelines, patients might benefit from being referred to specialist palliative care around or soon after the time of diagnosis, not only when they have reached the end-of-life phase of illness [63,64,65]. This is due to the progressive nature of neurological, cognitive, and personality changes, which may sometimes be rapid, especially when the disease is advanced. The early referral also allows the establishment of relationships with the providers and explore options for future care without the need for immediate decisions. It also facilitates continuity of care [63]. Referral to palliative care should be based on need, not on life expectancy [63]. Moreover, it is recommended that practitioners become familiar with palliative and hospice care resources available in their community [64]. This would help with educating patients and their families that involvement in palliative care services does not mean a state of hopelessness, abandonment, or no further treatment [64]. Although focusing on quality of life, palliative care is also concerned with the quality of dying [63]. Grief and bereavement support should also be offered to the family during the patient’s life and continuing after death [63].

Palliative care has also been historically underutilized in patients with glioblastoma [68]. Despite both the European Association of Neuro-Oncology (EANO) [3] and the National Institute for Health and Clinical Excellence (NICE) [69] recommending early referral to palliative and supportive care services, the actual provision of such care to people with primary brain tumors is scarce [70]. Recently, Shieh et al. [71] conducted a large population-based retrospective analysis (n = 1994) of the utilization of palliative care in patients with glioblastoma in Taiwan over a period of ten years. The authors reported that 34% of patients received palliative care. They further found that the survival of these patients who did receive palliative care was significantly longer than that of those who did not receive palliative care [71]. The results of this retrospective study need to be treated with caution, as individuals who live longer would have had more time to have palliative care services organized, which might explain why they received palliative care more often. However, the findings underscore the importance of including palliative care services for this group of patients.

## 8. Use of Services and Healthcare Professionals’ Perspective

Recently, initiatives on an international scale have been reported in efforts to gain insight into the current practice in the provision of supportive care and rehabilitation services. Halkett et al. [72] reported on a survey conducted with healthcare professionals in Australia, which aimed to document the supportive care available for patients with high-grade glioma and their caregivers and determine gaps in services. While many services were found to be available (such as cancer care coordinators, neuropsychologists, or radiation oncology nurses) to patients with high-grade glioma in Australia, the survey also highlighted areas in need of improvement, particularly access to psychosocial support and specialist allied health providers (such as dietitians, speech therapists, or exercise physiologists) [72]. The authors reported that a larger proportion of healthcare professionals had neuro-oncology multidisciplinary team meetings at their sites in metropolitan areas compared to rural locations [72]. Of note, only half of healthcare professionals reported that they could refer informal caregivers of patients with high-grade glioma to a support group [72]. The European Association for Neuro-Oncology (EANO) organized a survey assessing the level of inequalities in access to neuro-oncology supportive care and rehabilitation [28]. It was found that healthcare professionals who see only patients with glioma diagnoses refer patients to rehabilitation and supportive care less often as compared to those professionals who see people with a wider range of brain tumor diagnoses [28]. Participating healthcare professionals’ top reasons for not referring patients with unmet needs to appropriate healthcare services were a lack of options for referral for patients with malignant brain tumors, long waiting times, and staff shortages [28].

From the qualitative evidence on healthcare professionals’ perspective of glioblastoma treatment communication, we know that disease-specific symptoms such as cognitive issues, and a general sense of shock can present challenges to effective communication [73] and may affect patient and caregiver awareness of prognosis. Further, Philip et al. [74] documented the perspectives of medical, nursing, and allied health staff in relation to the supportive and palliative care needs of patients with malignant glioma. The results highlighted that patients with malignant gliomas face a different disease course compared to other cancers, including a sudden and unexpected deterioration of cognitive and physical function and fluctuating periods of deterioration and stability [74]. Healthcare professionals reported that balancing hope with reality and maintaining optimism was difficult in the face of disease progression and prognostic uncertainty [74]. They reported that traditional rehabilitation, which is aimed at patients with stable disease, is difficult when the prognosis and function can suddenly worsen [74]. It was also proposed that the focus of care shift from client-centered to family-centered in order to include the unmet needs of the informal caregivers and support their psychosocial concerns [74].

## 9. Patients’ Perspectives

Qualitative studies with individuals with glioblastoma can shed light on the potential reasons for the relatively low uptake of rehabilitation and supportive care services as seen from the patients’ perspective. Philip et al. [75] explored in-depth the perspectives of patients living and dying with diffuse glioma and found that patients had substantial needs, which they often did not share with others and consequently did not have addressed. Existential and psychosocial needs were particularly important to patients, but often remained unmet. One of the reasons reported by Philip et al. [75] includes patients’ fear and preference to remain independent. We recently reported a similar finding of individuals with brain tumors wanting to protect close others from the emotional burden and therefore not asking for support when needed [76]. Philip et al. [75] further noted that patients felt helpless in advocating for themselves even when already supported in a hospital. Patients and caregivers have also reported that they did not know who to contact in times of need [73]. Another reason reported by Philip et al. [75] for the difficulties in asking for relevant support was that patients emphasized their everyday, “here and now”, needs as opposed to acknowledging future needs. Such focus on the present moment has also been reported in other populations of individuals with brain tumors [77], where participants reported that making plans for the future was not a priority. Patients with high-grade gliomas also reported perceiving healthcare professionals as being more interested in immediate medical needs as opposed to being open to discussing future existential concerns or palliative care needs [75]. Furthermore, Gately et al. [78] examined the lived experience of long-term survivors of glioblastoma and found that they reflected on their previous activities, hobbies, and roles, and were redefining what was now possible and important to them to participate. They gathered these responses under the theme ‘Disconnection from past self: ‘*who I was*’’ [78]. For such a group of patients, who have exceeded the expectations of short life expectancy, rehabilitation might be helpful in redefining their identity and roles in life and facilitating participation in meaningful activities.

## 10. Implications for Development of Clinical Guidelines

Kim et al. [79] critically appraised published clinical practice guidelines for brain tumors and found that out of 11 clinical practice guidelines identified worldwide, only two included recommendations for rehabilitation interventions. The authors concluded that the components of rehabilitation included in both these guidelines, namely developed by the National Institute for Health and Clinical Excellence (NICE) [65] and the Australian Cancer Network (ACN) [63], were generic and described ambiguously [79]. Both guidelines recognized the importance of a comprehensive multidisciplinary approach with individually selected goals for therapy [79]. However, the information provided in both guidelines for the management of brain tumors was inconsistent and not detailed enough, making it difficult to synthesize the recommendations for rehabilitation. Future clinical guidelines should provide detailed information about the provision of each rehabilitation modality. Future clinical guidelines should also aim to make specific recommendations for interventions depending on the type of brain tumor, including glioblastoma. Regarding the implications for research to guide the development of future clinical guidelines, apart from conducting robust studies evaluating the effectiveness of rehabilitation interventions, qualitative findings on patients’, caregivers’, and clinicians’ perspectives should be incorporated to guide rehabilitation programs.

## 11. Conclusions and Future Directions

Rehabilitation therapy can play an integral role in the management of symptoms and complications in patients with glioblastoma. While there is limited rigorous scientific evidence available on inpatient as well as outpatient rehabilitation outcomes and optimal processes for patients with glioblastoma, the initial evidence summarized in this article points to beneficial effects. Overall, despite literature indicating patients’ needs and preferences for rehabilitation interventions [19], individuals with glioblastoma are not referred often enough [28,38,72]. We join the calls for further research into access to, and the benefits of, rehabilitation for this group of patients [37,38]. There is a pressing need to conduct high-quality research that would assess the effectiveness and benefits of rehabilitation and supportive care for patients with glioblastoma. Efforts are needed to design effective rehabilitation programs to match patient and caregiver needs [16]. Furthermore, there is a need to increase awareness and understanding of the positive outcomes that can be achieved through these services for neuro-oncology populations. Finally, there is a need for the development of clinical guidelines for rehabilitation practitioners to use when working with individuals with glioblastoma.

## Figures and Tables

**Table 1:** Summary of selected studies of physical rehabilitation in people with glioblastoma.

Author	Study Design	Population	Intervention	Outcomes
Hansen et al., 2019 [46]	Case study	A 54-year-old person with glioblastoma multiforme.	6-week multicomponent exercise program involving aerobic (cycling or treadmill), resistance, and balance training.	100% compliance with the exercise program. No adverse events. Improvements in aerobic power (↑24%), muscle strength (↑0–38%), standing balance (↑71%), walking ability (↑9%), and quality of life (EORTC-QLQ-C30, ↑8.6% in the domain “Global Health Status/QoL”).
Troschel et al., 2019 [50]	Case study	A 33-year-old person with glioblastoma multiforme.	21-month high-intensity exercise program involving aerobic (cycling and running), coordination, and resistance training.	No adverse events. Improved fitness diagnostic performance (↑32.8% in power to reach 75% of maximum heart rate over 17 months). Completed two marathons during the training period (in 210 min and 197 min, respectively). Averaged 43.7 metabolic equivalent of task hours per week (MET·h·wk^−1^).
Nowak et al., 2023 [49]	Feasibility/preliminary efficacy	25 people with glioblastoma scheduled to receive chemoradiotherapy.	Multicomponent exercise program for the duration of their chemoradiotherapy treatment involving aerobic (cycling, treadmill, rowing) and resistance exercise.	32% withdrawal prior to the study completion (8/25 participants). Low to high adherence (33–100%) and exercise dosage compliance (24–83%). No adverse events. Significant improvements in lower limb muscle strength (↑13.9%) and function (↑5.6%). No changes in body composition, fatigue, sleep, and quality of life.
Jost et al., 2023 [48]	Retrospective observational study	45 people with glioma (58% people with glioblastoma).	Multicomponent exercise program involving aerobic (cycling), coordination, and resistance training.	In 1828 training sessions, two minor epileptic events occurred. Improvement in watts per kg of body weight (↑11.5%) observed in a study subsample (n = 15). In retrospective analyses, people with glioblastoma who received the exercise had a longer median survival (24.1 months) compared to 325 people with glioblastoma who did not exercise (16.0 months), *p* ˂ 0.005.

The arrow (↑) indicates improvement.
